# *C*lamp-C*ru*shi*n*g versus *s*tapler *h*epatectomy for transection of the parenchyma in elective hepatic resection (CRUNSH) - A randomized controlled trial (NCT01049607)

**DOI:** 10.1186/1471-2482-11-22

**Published:** 2011-09-04

**Authors:** Nuh N Rahbari, Heike Elbers, Moritz Koch, Thomas Bruckner, Patrick Vogler, Fabian Striebel, Peter Schemmer, Arianeb Mehrabi, Markus W Büchler, Jürgen Weitz

**Affiliations:** 1 Department of General, Visceral and Transplant Surgery, University of Heidelberg, Germany; 2 Institute of Medical Biometry and Informatics, University of Heidelberg, Germany

## Abstract

**Background:**

Hepatic resection is still associated with significant morbidity. Although the period of parenchymal transection presents a crucial step during the operation, uncertainty persists regarding the optimal technique of transection. It was the aim of the present randomized controlled trial to evaluate the efficacy and safety of hepatic resection using the technique of stapler hepatectomy compared to the simple clamp-crushing technique.

**Methods/Design:**

The CRUNSH Trial is a prospective randomized controlled single-center trial with a two-group parallel design. Patients scheduled for elective hepatic resection without extrahepatic resection at the Department of General-, Visceral- and Transplantation Surgery, University of Heidelberg are enrolled into the trial and randomized intraoperatively to hepatic resection by the clamp-crushing technique and stapler hepatectomy, respectively. The primary endpoint is total intraoperative blood loss. A set of general and surgical variables are documented as secondary endpoints. Patients and outcome-assessors are blinded for the treatment intervention.

**Discussion:**

The CRUNSH Trial is the first randomized controlled trial to evaluate efficacy and safety of stapler hepatectomy compared to the clamp-crushing technique for parenchymal transection during elective hepatic resection.

**Trial Registration:**

ClinicalTrials.gov: NCT01049607

## Background

Hepatic resection forms the cornerstone of therapy for a variety of benign and malignant diseases of the liver [1]. While advances in patient selection, surgical technique, perioperative management and imaging tools reduced mortality substantially, morbidity of patients undergoing hepatic resection remains as high as 30-60% even at high-volume centers [2-7]. Due to the risk of intraoperative hemorrhage as well as postoperative morbidity (e.g. bile leakage, posthepatectomy hemorrhage), the period of actual transection of the liver parenchyma represents a crucial step during hepatic resection. Various studies could indeed demonstrate intraoperative hemorrhage as predictor of poor perioperative outcome in patients undergoing hepatic resection [1,8]. Although various devices have been developed to facilitate parenchymal transection with the ultimate aim to reduce intraoperative blood loss [9], a recent systematic review and meta-analysis failed to show a benefit of these tools compared to the simple clamp-crushing technique [10]. However, to the present there is no randomized controlled trial (RCT) evaluating the technique of stapler hepatectomy. Based on the well-established role of stapling devices in various surgical fields and their common use for division of hepatic veins and portal branches [11-13], vascular staplers may facilitate rapid division of the liver parenchyma with immediate sealing of vascular and biliary structures. In theory, these features enable hepatic resection to be being carried out with less intraoperative blood loss as well as lower operation time.

### Existing evidence and need for the trial

Several recent systematic review articles including a Cochrane review on transection techniques showed that there is a lack of high-level evidence, i.e. randomized controlled trials, on the use of stapling devices for parenchymal transection in elective hepatic resection [9,10,14,15]. While there are several articles on the technique of stapler hepatectomy as well as retrospective non-controlled studies [16-18], there is currently no RCT evaluating efficacy and safety of stapler hepatectomy compared to standard technique of parenchymal transection. The largest single-center experience on stapler hepatectomy has been reported from the Department of Surgery, University of Heidelberg. This analysis comprised 300 patients who underwent hepatic resection using the technique of stapler hepatectomy for transection of the parenchyma [19]. Even though this report and others suggests stapler hepatectomy to be rapidly feasible as well as effective and safe in controlling intraoperative blood loss [14,20], it's retrospective, non-randomized study design does not justify general recommendations.

### Aim of this trial

There is clinical uncertainty and ongoing discussion among liver surgeons regarding the optimal method of parenchymal transection in patients undergoing elective hepatic resection. While the clamp-crushing technique still represents the reference technique for routine liver resections [10], transection of liver parenchyma using vascular staplers may offer a new and safe technique potentially reducing intraoperative blood loss, operation time as well as peri-operative morbidity. As morbidity of patients undergoing hepatic resection remains high, approaches to reduce peri-operative complications are urgently required. Due to the lack of evidence it has to be evaluated, if the technique of stapler hepatectomy decreases intraoperative blood loss as a known predictor of peri-operative morbidity compared to the clamp-crushing technique. In case of comparable or more favorable secondary outcomes such as peri-operative mortality, transection time and need for re-interventions, this advantages would favor stapler hepatectomy as a routine technique for elective liver resections. As RCTs are generally considered to generate the most valid scientific evidence on a treatment's effects, the efficacy and safety of stapler hepatectomy needs to be evaluated in a randomized fashion.

## Methods/Design

### Trial population and patient recruitment

Patients scheduled for elective hepatic resection at the Department of General-, Visceral- and Transplantation Surgery, University of Heidelberg will be screened for enrollment into the trial. Patients meeting the eligibility criteria will be enrolled into the study. Informed consent is obtained at least on the day before surgery.

#### Subject Inclusion Criteria

Subjects matching the following criteria are eligible for inclusion into the clinical trial:

• Patients scheduled for elective hepatic resection

• Stapler hepatectomy and clamp-crushing feasible based on preoperative imaging

• Age equal or greater than 18 years

• Informed consent

#### Subject Exclusion Criteria

Subjects matching any of the following criteria must not be included into the clinical trial:

• Concomitant extraheptic resection planned

• Participation in concurrent intervention trials

• Expected lack of compliance

• Impaired mental state or language problems

### Study objectives and endpoints

The primary objective of this trial is to show that intraoperative blood loss during elective hepatic resection can be reduced by stapler hepatectomy as compared to the clamp-crushing technique.

The primary efficacy endpoint of the CRUNSH Trial is total intraoperative blood loss [ml], which is defined as blood loss from skin incision until closure of the skin. Intraoperative blood loss is measured according to the blood collected in the suction containers. Spilling water and ascites is subtracted. Furthermore, swabs are squeezed and their content will also be sucked and added to the fluid collected in the suction containers. To obtain a more precise estimate for the individual patient patient's individual transection area will be considered as a continuous covariate multivariate analysis. The transection area will be assessed using an imprint of the resected specimen on a paper sheet with a known density of 80 mg/m^2^.

To further evaluate efficacy and safety of stapler hepatectomy compared to the clamp-crushing technique, a set of general as well as surgical variables are documented as secondary endpoints [21]. The secondary endpoints of the trial are summarized together with their definition in Table 1.

**Table 1:** Secondary endpoints of the CRUNSH Trial

Secondary endpoint	Definition and assessment of outcomes
*Blood loss during liver transection [mL]*	Blood loss from beginning of parenchymal transection until minor oozing is stopped. To assess blood loss during liver transection the suction device will be connected to a new suction container for the period of actual hepatic transection.
Blood transfusion:	Administration of blood transfusions is documented for the intraoperative and postoperative period until 48 hours postoperatively. Documentation includes number of patients who received blood transfusions as well as amount of transfused packed red blood cells (PRBC) [units].
Operation time [min]:	Time from skin incision to placement of last skin staple/suture.
Liver transection time [min]:	Time from beginning to end of liver transection.
Duration of postoperative hospital stay [days]:	Time from day of operation to day of discharge.
Duration of ICU stay [days]:	Time on the Intensive Care Unit (ICU). Patients' stay in the recovery room and Intermediate Care (IMC) unit exceeding 24 hours is considered as ICU stay.
Morbidity:	The following predefined complications are documented within the CRUNSH Trial: Posthepatectomy hemorrhage [23]: Drop of hemoglobin level > 3 g/dl after the end of surgery compared to postoperative baseline level and/or any postoperative transfusion of PRBCs for a falling hemoglobin and/or the need for invasive re-intervention (e.g. embolization or re-laparotomy). Postoperative biliary leakage [24]: Presence of bile fluid (bilirubin level more than three times the serum level) in the abdominal cavity or drains on or after postoperative day or the need for reintervention (i.e. interventional drainage and/or relaparotomy due to bile fluid collections or biliary peritonitis). Further biliary complications: Biliary complications such as postoperative biliary stricture detected via ERCP and/or MRCP Intraabdominal fluid collection/abscess: Intraabdominal fluid collection detected on any imaging modality (e.g. ultrasound, CT scan) associated with abdominal discomfort/pain and/or elevation of infectious parameters. Posthepatectomy liver failure [25]: Increased INR or need of coagulation products (FFP, coagulation factors) to normalize the INR and increased serum bilirubin on or after postoperative day five. Pneumonia: Pulmonary infection with evidence of increased infection parameters (CRP > 2 mg/dl and/or leukocytes > 10 0000/ml) which are unlikely to be caused by a different pathologic process and evidence of pulmonary infiltrates on chest x-ray, requiring antibiotic therapy.
In-hospital mortality:	Death due to any reason within the patient's initial hospital stay.
Liver biochemical tests:	Serum levels of Alanine-Aminotransferase (ALT), Aspartate-Aminotransferase (AST), Gamma-glutamyl transferase (GGT), Quick's time/INR, Total Bilirubin, Albumin on postoperative day 1, 3 and 5.
Need for portal triad clamping:	Need for clamping of the hepatic pedicle to control intraoperative hemorrhage.
Resection margins	The proportion of patients with malignant tumors who have a positive resection margin will be documented.
Need for invasive re-interventions:	Invasive re-interventions such as placement of interventional drains, ERCP with stent placement and re-laparotomy within 30 days after the index operation or during patients' initial hospital stay.

### Standardisation of treatments

Patients' intra- and perioperative care will be standardized and kept identical except for the technique of hepatic transection.

Patients receive combined neuraxial and general anaesthesia. However, general anesthesia alone may be chosen, if neuraxial anesthesia is not considered safe by the executing anesthesiologist and lack of patient consent.

Following laparotomy via an extended right subcostal incision, a bilateral subcostal incision (with or without vertical midline extension or a reversed L-shaped incision from xiphoid to the tip of the twelfth right rib the abdomen is initially explored for extrahepatic disease. Intraoperative ultrasound of the liver is carried out regularly to exclude previously undetected lesions. Transection of the hepatic parenchyma is carried out under low central venous pressure (< 5 cmH_2_O). Central venous pressure (CVP) is lowered via fluid restriction and clamping of the inferior vena cava (IVC) [22]. If these prove insufficient to lower CVP to a level < 5 cmH_2_O, the Trendelenburg position and administration of nitrocompounds may be used in addition. Resections are carried out without use of hepatic inflow control (i.e. portal triad clamping). However, inflow control may be used by the surgeon, if considered necessary and will be documented as a secondary endpoint. Transfusion of PRBC within the CRUNSH Trial is standardized accoriding to the standards of care at the Department of General-, Visceral- and Transplantation and the Department of Anesthesiology, University of Heidelberg (Table 2).

**Table 2:** Transfusion triggers within the CRUNSH Trial

Risk profile	Minimum hemoglobin (conversion factor 0.621)
< 40 years **no **additional risk factors **no **organ function impairment	< 5.5 g/dl or 3.4 mmol/l

≥ 40 years **no **additional risk factors **no **organ function impairment	< 6 g/dl or 3.7 mmol/l

organ function impairment	< 7 g/dl or 4.3 mmol/l

coronary artery disease with no ischemia carotid artery stenosis with no ischemia history of transient ischemic attack	< 8 g/dl or 5.0 mmol/l

coronary artery disease with ischemia e.g. troponin elevation carotid artery stenosis with ischemia history of stroke	< 10 g/dl or 6.2 mmol/l

Argon beam coagulation is applied to stop minor oozing once resection is completed and sealants may be used, if deemed necessary by the executing surgeon. After hemostasis is considered secure, easy-flow drains are placed in the subphrenic and subhepatic space and the abdomen is closed in a standardized manner.

### Trial interventions

#### Group A: Clamp-crushing technique

The transectional line is marked and the liver capsule is cauterized. The liver parenchyma is then crushed stepwise using a regular Pèan clamp. Vessels of less than 2 mm in diameter are coagulated with the irrigated bipolar forceps. The remaining vessels are clipped or ligated. The hepatic veins and the portal triad are divided using sutures or the Autosuture Endo Gia™ Universal Stapler and Endo Gia™ Universal Angulating 45 mm loading units with 2.5 mm staples (Covidien).

#### Group B: Stapler hepatectomy

The transectional line is marked and the liver capsule is cauterized. For subsequent transection of the hepatic parenchyma, the liver tissue is fractured with a vascular clamp in a stepwise fashion and subsequently divided using the Autosuture Endo Gia™ Universal Stapler and Endo Gia™ Universal Straight 60 mm loading units with 2.5 mm staples (Covidien). The hepatic veins and the portal triad are divided using the Autosuture Endo Gia™ Universal Stapler and Endo Gia™ Universal Angulating 45 mm loading units with 2.5 mm staples (Covidien).

### Trial implementation

Patients scheduled for elective hepatic resection are screened consecutively for inclusion into the trial. All patients screened for the CRUNSH Trial are documented in the screening log. Patients meeting the inclusion criteria are enrolled in the trial. Informed consent has to be obtained at least on the day prior to surgery. Study visits within the CRUNSH Trial are displayed in Table 3.

**Table 3:** Flow chart of the CRUNSH Trial

	Screening	Intervention	Follow Up		
	**Visit 1** **(up to 5 days before surgery)**	**Visit 2** **(day of surgery)**	**Visit 3** **(POD 1)**	**Visit 4** **(POD 3)**	**Visit 5** **(POD 7 or day of discharge)**

**Eligibility criteria Informed consent**	·				
**Medical history**	·				
**Demographics**	·				
**Physical examination**	·				
**Laboratory tests**	·		·	·	·
**Randomization**		·			
**Trial intervention**		·			
**Intraoperative parameters**		·			
**Postoperative parameters**			·	·	·

POD, Postoperative day

### Methods against Bias

#### Randomization

Patients are screened consecutively and all eligible patients are included into the trial. In order to achieve comparable groups and ensure allocation concealment patients are randomly allocated to the study groups. A block randomisation list is generated by the Institute for Medical Biometrics and Informatics (IMBI) applying SAS (SAS™ Version 9.1., SAS Institute Inc., Cary, USA). Randomization is carried out intraoperatively in case resectability is given. Randomization is carried out using opaque and sealed envelopes that are consecutively numbered. Block randomization will be performed for each center to achieve equal group sizes per center. The details of randomization will be kept in safe and confidential. Subjects withdrawn from the trial retain their identification codes (e.g. randomization number). New subjects receive a new identification code.

#### Blinding

Patients are blinded for the study intervention. Blinding of the surgeon and people in the operating room is not feasible. Therefore a third party blinded to patients' allocated treatment group assesses postoperative outcomes.

#### Standardization of care

To assure comparable treatment of patients, all surgeons who participate in this trial, will be instructed on both study interventions. Intra-and perioperative care is standardized. In particular, central venous pressure (CVP) will be lowered to < 5 mmHg for the period of parenchymal transection and resections in both study arms will be performed without routine use of the vascular control. Furthermore, the area of resection will be included in the multivariate analysis as a continuous covariate.

### Sample size

The sample size is based on the primary outcome parameter and the primary analysis. Internal observations (Department of Surgery, University of Heidelberg) showed a mean intraoperative blood loss of 700 ml for patients undergoing stapler hepatectomy with a sample standard deviation of about 550 ml [19]. To detect a clinically relevant absolute difference reduction in intraabdominal blood loss of 280 ml with significance α = 5% and a power of (1-β) = 80% n = 122 patients have to be randomized in the study (n = 61 patients per group) using two-sided t-test. Considering an estimated intraoperative drop-out rate of about 10% (e.g. unexpected death prior to beginning of transection, protocol violations) eight additional patients will be randomized and the total sample size accounts for n = 130 patients (n = 65 patients per group).

### Statistical analysis

Statistical methods are used to assess the quality of data, homogeneity of treatment groups, endpoints and safety of the two intervention groups. The confirmatory analysis is performed on the basis of an intention-to-treat (ITT) population and with respect to ITT principles.

Categorical data are summarized by means of absolute and relative frequencies (count and percent). Continuous data are presented by means of the following summary statistics: the number of observations, median, minimum, median and maximum. Wherever appropriate, data are visualized by box-whisker plots or histograms. The primary efficacy endpoint is amount of total intraoperative blood loss. The underlying two-sided null-hypothesis is that both study interventions lead to similar intraoperative blood loss:

$${\text{H}}_{0}:\mu 1-\mu 2=0$$

The alternative is that one intervention performs better than the other:

$${\text{H}}_{1}:\mu 1-\mu 2\neq 0$$

A confirmatory intention-to-treat analysis (2-sided test), including all patients as randomized, is performed on the amount of intraoperative blood loss between the two groups. Analysis of covariance (ANCOVA) is used to detect possible treatment differences with intraoperative blood loss as dependent variable, area of resection and CVP during transection as continuous covariates and type of intervention as factor. The sample size calculation is based on a two-sided t-test, and it can be assumed that evaluation with analysis of covariance has the same or even higher power.

Secondary endpoints will be analyzed in an exploratory way, using appropriate statistical methods based on the underlying distribution of the data. All analysis will employ SASα Version 9.1.

### Data management and quality assurance

The investigator or a designated representative enters all protocol-required information in the case report form (CRF). The CRF should be completed as soon as possible after the information is collected, preferably on the same day when a trial subject is seen for an examination, treatment, or any other trial procedure. The reason for missing data should be provided. The investigator is responsible for ensuring that all sections of the CRF are completed correctly and that entries can be verified in accordance with the source data.

The completed CRF must be reviewed and signed by the investigator named in the trial protocol or by a designated sub-investigator. The principle investigator will retain originals of all CRF at the end of the trial

Monitoring within the CRUNSH Trial is carried out by an independent investigator at the Department of Surgery, University of Heidelberg, who is not involved in the trial and in completing the CRFs. The basic data of all participating patients are completely checked, i.e. existing patient, patient number, initials, the availability of signed informed consent. For a proportion of 10% of the study participants (randomly selected) a complete check of all data in the CRF (i.e. a 100% clinical source data verification; SDV) is carried out. The extent of further SDV is dependent on the quality of the data and occurrence of protocol violations.

### Ethical and legal considerations

The study is conducted in agreement with either the Declaration of Helsinki (Tokyo, Venice, Hong Kong, Somerset West and Edinburgh amendments) or the laws and regulations of the country, whichever provides the greatest protection of the patient. The protocol has been written, and the study will be conducted according to the ICH Harmonized Tripartite Guideline for Good Clinical Practice (ref: http://www.ich.org/fileadmin/Public_Web_Site/ICH_Products/Guidelines/Efficacy/E6_R1/Step4/E6_R1__Guideline.pdf).

The trial protocol is approved by the local independent ethics committee of the University of Heidelberg approved the trial protocol, the patient information and informed consent sheet.

The CRUNSH Trial is registered at the ClinicalTrials.gov protocol registration system (NCT01049607).

All patients will be informed of the aims of the study, the possible adverse events, the procedures and possible hazards to which he/she will be exposed to, and the mechanism of treatment allocation. They will be informed as to the strict confidentiality of their patient data, but that their medical records may be reviewed for trial purposes by authorized individuals other than their treating physician. The signed consent document is kept by the investigator. A copy of the signed consent document is handed out to the subject or the subject's legally authorized representative.

It will be emphasized that the participation is voluntary and that the patient is allowed to refuse further participation in the study whenever he/she wants. This will not influence the patient's subsequent care. Documented informed consent must be obtained for all patients included in the study before they are registered or randomized in the study. This must be done in accordance with the national and local regulatory requirements.

It is the responsibility of the investigator to maintain patient's confidentiality. During the trial, patients will be identified solely by means of their initials, age and individual identification code (screening number, randomization number). Trial findings will be stored in accordance with local data protection law/ICH GCP Guidelines and will be handled in strictest confidence. For protection of these data, organizational procedures are implemented to prevent distribution of data to unauthorized people.

The investigator will maintain a personal subject identification list (screening numbers with the corresponding subject names) to enable records to be identified.

## Abbreviations

**ALT**: Alanine aminotransferase; **AST**: Aspartate aminotransferase; **ANCOVA**: Analysis of covariance; **CRF**: Case Report Form; **CV**: Curriculum vitae; **CVP**: Central venous pressure; **GCP**: Good Clinical Practice; **GGT**: Gamma glutamyl transpeptidase; **ICH**: International Conference on Harmonization of Technical Requirements for Registration of Pharmaceuticals for Human Use; **INR**: International normalized ratio; **IVC**: Inferior vena cava; **POD**: Postoperative day; **PRBC**: Packed red blood cells; **RCT**: Randomized controlled trial; **SDV**: Source Data Verification

## Competing interests

The authors declare that they have no competing interests.

## Authors' contributions

This study was designed by NNR, HE and JW who also wrote the article. TB performed the sample size calculation and planned the statistical analyses. MK, PV, FS, PS, AM and MWB are involved in trial implementation and critically revised the manuscript. All authors have read and approved the manuscript.

## Pre-publication history

The pre-publication history for this paper can be accessed here:

http://www.biomedcentral.com/1471-2482/11/22/prepub
